# Isolation of coronavirus disease 2019 (COVID-19) patients in cohorted wards or single-patient rooms? Advantages and disadvantages

**DOI:** 10.1017/ice.2020.1425

**Published:** 2021-01-11

**Authors:** Manon D. van Dijk, Diana van Netten, Juliëtte A. Severin, Ed F. van Beeck, Margreet C. Vos

**Affiliations:** 1 Department of Medical Microbiology and Infectious Diseases, Erasmus MC University Medical Centre Rotterdam, The Netherlands; 2 Department of Public Health, Erasmus MC University Medical Centre Rotterdam, The Netherlands

A sudden and rapid influx of patients in acute care occurred in March 2020 in the Netherlands, when the new coronavirus disease 2019 (COVID-19)–specific infection prevention policies were not fully developed. Scarcity of personal protective equipment (PPE), nearly reaching the maximum capacity of hospitals, and the enormous workload for healthcare workers (HCWs), forced many hospitals to decide to cohort patients with COVID-19. Other considerations in choosing cohorting were hospital design with predominantly multiple-occupancy rooms, lack of capacity to isolate each patient in a single-patient room with doors closed, and expecting to provide more efficient patient care.

In cohorts, the doors of COVID-19 patients rooms remain open. These rooms can either be multiple-occupancy or single-patient rooms or a mix of both, but in all cases, the rooms, including enclosed parts on the ward, are used to care for proven COVID-19 patients. The entire cohort ward is considered contaminated; therefore, only controlled entry to the ward is allowed.^1^ Consequently, PPE has to be worn when entering the cohort ward and during whole shifts. PPE has to be changed after wearing it for 3–6 consecutive hours with different patients.^2,3^ Mainly, since the effectiveness of surgical face masks will be reduced after this time, but also because of eating, drinking, and bathroom use, which should take place outside the ward. All PPE should be removed when leaving the cohort ward. On wards where COVID-19 patients are only isolated in single-patient rooms with doors closed, only that patient room is considered contaminated. Non–COVID-19 patients in other (adjacent) rooms at the same ward can be cared for without using extra PPE. Here, we describe our investigation of whether cohorting or isolation in single-patient rooms with doors closed saves PPE. We highlight the advantages and disadvantages of organizing patient care in cohort wards or in single-patient rooms with doors closed.

PPE use was observed in the Erasmus MC University Medical Centre Rotterdam (Erasmus MC), during the first peak of COVID-19 in the Netherlands, in March–April 2020. COVID-19 patients were cared for in single-patient rooms with doors closed; we did not create cohorts. This type of isolation was most obvious because our hospital consists of 100% single-patient rooms for adults. Trained medical students counted the number of HCWs who entered single-patient rooms, while wearing a full PPE set, during 4 busy hours in the morning. One PPE set (ie, gloves, face mask, eye protection, gown) to be used for COVID-19 patients, was considered equivalent to 1 HCW entering the room.

The use of 278 PPE was observed on 2 general wards (ie, wards 1 and 2) and 3 ICUs (ie, wards 3, 4, and 5) between April 14, 2020, and May 1, 2020. The frequencies of PPE use observed for 1 patient in 4 hours were 7 for ward 1, 8 for ward 2, 7 for ward 3, 9 for ward 4, and 8 for ward 5. The data show no large differences in PPE use between a general ward and an intensive care unit (ICU). On average, 8 PPE (standard deviation [SD], 0.7) were used to care for 1 patient in 4 hours.

According to the Dutch guideline, every 3 hours, a face mask has to be changed while working in a cohort due to expiration.^2^ Therefore, we calculated PPE use when caring for 1 patient in 3 hours, which was 6 PPE. When we multiply this number with the total number of patients hospitalized on the ward during the observation, we were able to estimate the total PPE use in the observed ward. This value could then be used as a break-even point for installing a cohort situation. The PPE use in 3 hours for the entire ward was 234 PPE (39 admitted patients) for wards 1 and 2; 198 PPE (33 admitted patients) for ward 3; 132 PPE (22 admitted patients) for ward 4; and 36 PPE (6 admitted patients) for ward 5. On average, 167 PPE were used in a cohort every 3 hours.

When a cohort uses <6 PPE per patient per 3 hours, the hospital will save PPE compared to isolation in single-patient room setting with doors closed. These calculated cohort situations suggest that, with small groups of patients, PPE use is more likely to exceed the break-even point faster, which makes isolation in single-patient room with doors closed more efficient. However, cohorting would probably be more efficient when isolating larger patient groups.

Apart from efficiency, several other factors can guide the choice for COVID-19 care in cohorted wards or single-patient rooms. Therefore, we have presented the advantages and disadvantages of both types of isolation, based on literature and expert opinion in Table 1. By including published literature and expert opinion, we have provided a complete overview of the issues to consider when deciding on the isolation organization of COVID-19 patients in the hospital. One limitation of our study is that we may have overestimated our observations because we observed PPE use during the busiest hour of the morning. We expect that less PPE is used during other working hours.

**Table 1.** Advantages and Disadvantages of Cohorting Versus Single-Patient Room Care

Item Under Consideration	Cohort		Single-Patient Room With Closed Doors	
	Advantage	Disadvantage	Advantage	Disadvantage
Capacity of the ward	Multiple-occupancy bedrooms have a higher bed-capacity compared to single-patient rooms in a comparable building surface or area.^a^	**…**	…	Isolation capacity is limited by availability of single-patient rooms.^a^
Flexibility per room	…	All patient rooms in the cohort are contaminated and not available for non-COVID-19 care.^a^	Flexibility per room is high, in terms of use for isolation (proven and suspected) or non-COVID-19 care.^a^	**…**
Risk of patient-to-patient transmission of microorganisms other than COVID-19	…	Multiple-occupancy rooms are a risk factor for nosocomial transmission.^4^	Single rooms have lower risk for nosocomial transmission.^4^ HCW are more likely to perform hand hygiene in case of single rooms.^5^	**…**
Use of PPE	It is more convenient to care for more patients without donning and doffing PPE per patient.^a^ Less donning and doffing means less chance of self-contamination with microorganisms.^6^	PPE is needed for all persons entering the ward.^a^ Wearing PPE for a prolonged period may increase inadequate use of PPE (eg, touching mask).^3^ HCW have to change PPE after 3 h because of expiration of face masks but also because of eating, drinking and using the bathroom outside of the ward.^2^	Not all personnel at the COVID-19 ward have to wear PPE.^a^	Donning and doffing of PPE per patient rooms, increases the chance of self-contamination.^6^
Working conditions HCW	**…**	Wearing PPE (ie, gloves, a face mask, eye protection and a gown) all day is uncomfortable and demanding.^7^	PPE only in patient rooms, results in more comfort and better working conditions for HCWs because wearing time is restricted to patient care moments.^a^	**…**
Safe patient care	Clear overview on all patients as doors are open or the ward has an open design.^a^	Allocation of patients depends on the accuracy of PCR or other test results (ie, false-positive PCR results).^a^	Patients awaiting their results of COVID-19 tests, are not exposed to positive COVID-19 patients.^8^	The threshold for HCWs to enter the patient room is higher, which can result in less HCW–patient contact.^9^
Patient well-being	…	It can have a major impact on a patient when other patients nearby suddenly become very ill or pass away.^a^	Patients will sleep better, which most likely benefits to a faster recovery and a shorter length of stay.^10^	Patients may feel lonely because they are separated from other patients.^1,10^
Visitors	**…**	Visitors have to adhere to PPE regulations on entering the ward. Communication is difficult when wearing PPE, which makes it challenging to give instructions on the correct use of PPE.^a^ No. of visitors adds up to no. of persons present and possibly exposed at the cohort.^a^	Visitors can enter the ward freely, receive instructions clearly, and only wear PPE in the patient room.^a^ More visitors can be allowed as long as there is no shortage of PPE and instruction is possible.^a^	**…**
Ease of cleaning and disinfection	**…**	All patients have to be discharged before the entire ward can be completely cleaned and disinfected.^a^	The patient room can be cleaned and disinfected separately in time and place from other rooms.^a^	**…**
Logistics of supply, storage and disposal of materials and waste	**…**	Sterile/clean supplies and equipment have to be stored outside of the cohort area.^a^ A special anteroom is needed for donning and doffing of PPE.^a^	Daily supplies are kept in the patient room. Everything is discarded after discharge of the patient.^a^ Donning and doffing of PPE by the HCW is done before entering and leaving the patient room.^a^	**…**

Note. HCW, healthcare workers; PPE, personal protective equipment; PCR, polymerase chain reaction.

a Expert opinion/ experience.

In conclusion, before choosing isolation in single-patient rooms with doors closed or establishing a cohort, it is important to consider the expected usage of PPE with respect to the number of COVID-19 patients as well as the specific advantages and disadvantages of the options.
